# Psoriasis Flare-Up After COVAXIN BBV152 Whole Virion Inactivated Vaccine

**DOI:** 10.7759/cureus.22311

**Published:** 2022-02-17

**Authors:** DJ L Infimate, Deepak Yumnam, Santosh S Galagali, Ankita Kabi, Nidhi Kaeley

**Affiliations:** 1 Emergency Medicine, All India Institute of Medical Sciences, Rishikesh, IND; 2 Dermatology, All India Institute of Medical Sciences, Rishikesh, IND; 3 Emergency Medicine (Anesthesiology), All India Institute of Medical Sciences, Rishikesh, IND

**Keywords:** covid, vaccine, trigger, flare-up, psoriasis

## Abstract

Psoriasis is an inflammatory skin condition with a chronic relapsing course that can negatively impact a patient's quality of life. Various triggering factors can cause the flare-up of psoriasis, which also include vaccination. The most common vaccine associated with this is influenza. In this global pandemic of coronavirus disease 2019 (COVID-19), emergency authorization for mass vaccination has been adopted by many countries in the world. Psoriasis flare has been reported after the Pfizer COVID-19 vaccine and CoronaVac vaccine. Currently, both the virus-causing disease and the vaccines are still being studied owing to their dynamicity. We report a case of a 21-year-old gentleman with chronic plaque psoriasis of three years, who developed generalized pustular psoriasis eruption after administration of the first dose of COVAXIN. To the currently available literature, this was the first case of this complication associated with COVAXIN.

## Introduction

Psoriasis is a chronic, immunologically mediated, inflammatory skin disease affecting 1-3% of the world’s population. The classic lesion of psoriasis is characterized by well-defined, erythematous, indurated scaly plaques, with predilection of extensor surfaces. It can involve the scalp and nails also. There are different types of psoriasis including plaque-type, pustular, guttate, inverse, erythrodermic, nail, and psoriatic arthritis. Psoriasis is an autoimmune disease mediated by T lymphocytes and is typically a lifelong inflammatory condition with a fluctuating course of exacerbation and remission of lesions. It may be aggravated by infection, emotional stress, genetics, local trauma, and environmental factors [1]. Vaccination is an uncommon triggering factor for psoriasis flare though it has been reported previously. The exact etiological association between these two is not known. However, in the case of influenza vaccine-induced psoriasis, T-helper (Th1 and Th17) immunologic responses are thought to be the possible underlying mechanism [2].

COVAXIN is a whole virion inactivated vaccine developed by Indian pharmaceutical company Bharat Biotech in collaboration with the Indian Council of Medical Research and the National Institute of Virology (NIV). It is now under phase 3 clinical trial. It mainly contains 6 µg of the whole virion inactivated severe acute respiratory syndrome coronavirus 2 (SARS-CoV-2) antigen (strain: NIV-2020-770), and the other inactive components such as 250 µg aluminum hydroxide gel, 15 µg toll-like receptor (TLR) 7/8 agonist (imidazoquinolinone), 2.5 mg 2-phenoxyethanol, and phosphate buffer saline up to 0.5 ml [3].

The ongoing coronavirus disease 2019 (COVID-19) pandemic has a severe impact on the treatment of various chronic inflammatory diseases including psoriasis, concerning follow-up and compliance to the treatment, and consultations resulting in exacerbation of the disease. The patients may have modified or even discontinued their treatment due to misinformation and ignorance.

We here present the first (to the currently available literature) psoriasis patient who presented with a generalized pustular psoriasis flare acutely after the first dose of COVAXIN administration.

## Case presentation

A 21-year-old man presented to the emergency department with psoriasis pustular flare-up after four days of the first dose of Bharat Biotech inactivated vaccine (COVAXIN). He was a known case of chronic plaque psoriasis for eight years and was on topical steroids for the last two years, which he was taking as and when advised with adequate control of the disease. His body surface area (BSA) involved was calculated by the Wallace rule of nines, and psoriasis area and severity index (PASI) were 3-4% and 1.6, respectively, before the vaccination. He reported complaints of pus-filled lesions all over the body sparing his palm and soles, associated with burning sensation, but there was no itch or pain. There was no history of infection, stress, or any intake of steroids or immunosuppressants.

**Figure 1:**
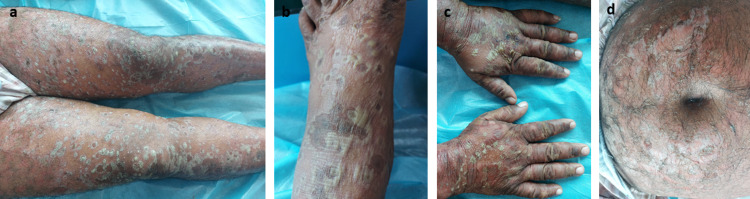
Clinical presentation of the patient on admission showing diffuse pustules over (a) lower limb, (b) feet, (c) hands, and (d) abdomen.

The psoriasis flare started initially as erythema associated with a burning sensation over the shoulder and gradually progressed to the abdomen, back, and bilateral upper and lower limbs over the next four days. The character of predefined psoriatic plaque (induration with scaling) got lost during this course of time. Physical examination revealed diffuse erythema, desquamation, and coalescing pustules over the entire body with a BSA of 40-45% (Figure 1).

Investigations revealed an increased total leukocyte count of 22.56 x 109/L with neutrophil and lymphocyte differentiation of 79% and 15%, respectively, elevated C-reactive protein at 154.9 mg/L, and procalcitonin of 0.11 ng/mL (Table 1).

**Table 1:** Investigations of the patient. RT-PCR, reverse transcription-polymerase chain reaction.

S. No.	Investigations	Values
1.	Complete blood count	
	Hemoglobin	11.33 g/dL
	Total leucocyte count	22,560/mm^3^
	Neutrophils	79%
	Lymphocytes	15%
	Monocytes	4%
	Eosinophils	1%
	Basophils	1%
	Hematocrit	45%
	Platelet count	3,56,000/mm^3^
2.	C-reactive protein	154.9
3.	Procalcitonin	0.11
4.	Blood culture	No growth within 48 hours of incubation
5.	Pus culture	No growth within 48 hours of incubation
6.	COVID-19 RT-PCR	Negative

Based on the history and clinical findings, he was diagnosed with generalized pustular psoriasis exacerbation associated with the COVAXIN vaccine. The patient was given acitretin 25 mg once daily. The patient responded well to acitretin with no new pustules formation and was discharged after one week of hospital admission.

## Discussion

Treatment of various chronic inflammatory disorders including psoriasis has been affected drastically during the COVID-19 pandemic. Irregular consultations and drugs unavailability resulted in exacerbations of psoriasis in many patients. Furthermore, the question is whether psoriasis patients intend to receive COVID-19 vaccination as the psoriasis flare is well known with influenza vaccine attributing it to a T-helper (Th17)-mediated immunological response [4,5]. A study conducted by Sotiriou et al. demonstrated that patients with psoriasis were willing to receive COVID-19 vaccination compared with the control group, which consisted of patients with other skin diseases on immunosuppressive treatment (OR: 1·32, 95% CI: 1·28-1·36). While 21% of 713 psoriasis patients declared fear of potential post-vaccination flare of their skin disease to be holding them back from receiving a COVID‐19 vaccine [6].

Psoriasis flare is also reported with other COVID-19 vaccines such as Pfizer-BioNTech BNT16B2b2 mRNA vaccine and CoronaVac vaccine (Sinovac Biotech) [7,8]. In a Greek cohort, 14 patients with psoriasis reported generalized papulosquamous rash post-vaccination with three European Medicines Agency‐approved SARS‐CoV‐2 vaccines (Pfizer mRNABNT162b2, Moderna mRNA-1273, and AstraZeneca‐Oxford AZD1222), which are either mRNA vaccines or adenovirus-based vaccines. It was mostly reported after the second dose of different vaccines with no statistical difference in the PASI score amongst the different vaccine groups. However, nine of these patients had left the treatment of psoriasis for one year and five patients were on topical treatment [9].

COVAXIN uses a different technology, namely, the whole virion inactivated Vero cell-derived platform [10]. It was approved by the Central Drugs Standard Control Organisation (CDSCO) for emergency use on 3rd January 2021. The interim results of the phase 3 trial were published recently and the most common adverse event reported was pain at the injection site, followed by headache, fever, and fatigue. No severe or life-threatening solicited adverse events were reported [3].

Other non-COVID vaccines which have reported psoriasis flare include influenza (H1N1), pneumococcal pneumonia, and yellow fever vaccine [4,5,11,12]. A new onset guttate psoriasis has also been reported after intravesical immunotherapy of Bacillus Calmette-Guerin (BCG) [13]. Vaccination as a triggering factor is rare; however, since worldwide vaccination is going on, medical professionals should be more vigilant about the possible adverse effects and hence their management. Moreover, to date, there is no well-described association with the COVID-19 vaccines.

The National Psoriasis Foundation (NPF) COVID-19 task force has developed guidance statements to promote optimal management of psoriatic disease during this pandemic. They stated that patients with psoriasis who do not have any contraindication to vaccination should receive an mRNA-based COVID-19 vaccine and systemic medications for psoriasis or psoriatic arthritis [14]. However, several systemic treatments for skin disorders have been linked to reduced vaccine-induced protective immunity [15]. Besides, the excipients contained in the vaccine may also cause anaphylaxis. Polyethylene glycol in the Pfizer COVID-19 vaccine is reported to cause anaphylaxis [16]. In this current scenario, such patients should be more vigilant.

Weighing the potential benefits and risks, many authors recommend COVID-19 vaccination for all psoriatic patients including those on immunosuppressant drugs. Though there is no guideline regarding the monitoring of the patient during the vaccination period, monitoring and follow-up should be done during and after the vaccination. We present the first case report of COVAXIN-associated flare-up of psoriasis. Considering our case and other few case reports, a guideline should be considered in such patients, and since there is a lack of data on the safety of novel mRNA COVID-19 vaccines, further study is needed.

## Conclusions

Considering our case and other few case reports, health professionals should be vigilant about the possible adverse effects of COVID-19 vaccines, and patients are advised to report to healthcare services promptly to prevent worsening of clinical condition. As extensive and rapid vaccination is going on, vaccine recipients should be monitored and followed up after vaccination since there is a lack of data on the safety of novel mRNA COVID-19 vaccines.

## References

[1] Gudjonsson JE, Elder JT (2019). Psoriasiform disorders. Fitzpatrick’s Dermatology.

[2] Gunes AT, Fetil E, Akarsu S, Ozbagcivan O, Babayeva L (2015). Possible triggering effect of influenza vaccination on psoriasis. J Immunol Res.

[3] Ella R, Reddy S, Blackwelder W (2021). Efficacy, safety, and lot-to-lot immunogenicity of an inactivated SARS-CoV-2 vaccine (BBV152): interim results of a randomised, double-blind, controlled, phase 3 trial. Lancet.

[4] Shi CR, Nambudiri VE (2017). Widespread psoriasis flare following influenza vaccination. Vaccine.

[5] Shin MS, Kim SJ, Kim SH, Kwak YG, Park HJ (2013). New onset guttate psoriasis following pandemic H1N1 influenza vaccination. Ann Dermatol.

[6] Sotiriou E, Bakirtzi K, Papadimitriou I, Paschou E, Vakirlis E, Lallas A, Ioannides D (2021). COVID-19 vaccination intention among patients with psoriasis compared with immunosuppressed patients with other skin diseases and factors influencing their decision. Br J Dermatol.

[7] Krajewski PK, Matusiak Ł, Szepietowski JC (2021). Psoriasis flare-up associated with second dose of Pfizer-BioNTech BNT16B2b2 COVID-19 mRNA vaccine. J Eur Acad Dermatol Venereol.

[8] Onsun N, Kaya G, Işık BG, Güneş B (2021). A generalized pustular psoriasis flare after CoronaVac COVID-19 vaccination: case report. Health Promot Perspect.

[9] Sotiriou E, Tsentemeidou A, Bakirtzi K, Lallas A, Ioannides D, Vakirlis E (2021). Psoriasis exacerbation after COVID-19 vaccination: a report of 14 cases from a single centre. J Eur Acad Dermatol Venereol.

[10] Thiagarajan K (2021). What do we know about India's Covaxin vaccine?. BMJ.

[11] Yoneyama S, Kamiya K, Kishimoto M, Komine M, Ohtsuki M (2019). Generalized exacerbation of psoriasis vulgaris induced by pneumococcal polysaccharide vaccine. J Dermatol.

[12] de Barros MH, Avelleira JC, Mendes KA (2019). Impact of yellow fever vaccine on patients with psoriasis: preliminary results. An Bras Dermatol.

[13] Hung CT, Wang WM, Tsao CW, Chiang CP (2012). New-onset guttate psoriasis following intravesical immunotherapy of Bacillus Calmette-Guerin. Dermatol Sin.

[14] Gelfand JM, Armstrong AW, Bell S (2021). National Psoriasis Foundation COVID-19 Task Force guidance for management of psoriatic disease during the pandemic: version 2—advances in psoriatic disease management, COVID-19 vaccines, and COVID-19 treatments. J Am Acad Dermatol.

[15] Speeckaert R, Lambert J, Puig L, Speeckaert M, Lapeere H, De Schepper S, van Geel N (2021). Vaccinations in patients receiving systemic drugs for skin disorders: what can we learn for SARS-Cov-2 vaccination strategies?. Drugs R D.

[16] Sellaturay P, Nasser S, Islam S, Gurugama P, Ewan PW (2021). Polyethylene glycol (PEG) is a cause of anaphylaxis to the Pfizer/BioNTech mRNA COVID-19 vaccine. Clin Exp Allergy.

